# Risk assessment in aortic aneurysm repair by medical specialists versus the American College of Surgeons National Surgical Quality Improvement Program risk calculator outcomes

**DOI:** 10.1177/20480040211006582

**Published:** 2021-04-08

**Authors:** Jan van Schaik, Tessa M Hers, Carla SP van Rijswijk, Maaike S Schooneveldt, Hein Putter, Daniël Eefting, Joost R van der Vorst

**Affiliations:** 1 Department of Surgery, Leiden University Medical Centre, Leiden, The Netherlands; 2 Department of Radiology, Leiden University Medical Centre, Leiden, The Netherlands; 3 Department of Anaesthesiology, Leiden University Medical Centre, Leiden, The Netherlands; 4 Department of Medical Statistics and Bioinformatics, Leiden University Medical Centre, Leiden, The Netherlands

**Keywords:** Risk assessment, National Surgical Quality Improvement Program risk calculator, vascular surgeons, interventional radiologists, anaesthesiologists

## Abstract

**Objective:**

The aim of this online clinical vignette-based survey study was to compare risk assessments by vascular surgeons, anaesthesiologists and interventional radiologists involved in treating patients with aortic aneurysms in the Netherlands with the NSQIP risk calculator outcomes.

**Methods:**

Participants, recruited using purposive sampling, provided their estimation of the likelihood of postoperative complications and events following aortic surgery in five fictional cases. These cases were subsequently scored using the NSQIP calculator. The risk assessments were statistically analysed using the ANOVA and student t-test.

**Results:**

All participating specialists i.e. twelve vascular surgeons, ten interventional radiologists and ten anaesthesiologists completed the survey. In the vast majority of outcomes and vignettes, no significant differences were found between various specialists, whereas significant differences were found between the NSQIP risk calculator outcomes and the combined risk assessments of the specialists. Overall, specialist risk assessments differ from the NSQIP, but neither particularly higher nor lower compared to the risk calculator.

**Conclusions:**

Risk assessment by vascular surgeons, anaesthesiologists and interventional radiologists differs significantly with NSQIP risk calculator outcomes, within the framework of both endovascular and open aortic aneurysm repair. Based on these results, implementing the NSQIP risk calculator in preoperative workup could be of added value in both patient planning as well as adequately informing patients for obtaining consent.

## Introduction

Aneurysmal aortic disease is common in the Western world, with prevalence differing among countries, age and gender.^1^ The total worldwide mortality rate of aortic aneurysms and dissection was reported in 2010 as 2.78 per 100.000 inhabitants. Apart from the size of the aneurysm, the decision to surgically treat an aneurysm is based on individual patient factors such as anatomy, comorbidities and predicted clinical outcome.^2^

There are several techniques to treat aortic aneurysms. These include standard endovascular aneurysm repair (EVAR), complex endovascular repair (e.g. fenestrated or branched EVAR) and open surgical repair (OSR).^3,4^

Treatment of aortic aneurysms imposes risks of potentially severe complications. Besides anatomic aneurysm characteristics, patient condition, frailty and comorbidities all affect perioperative mortality, morbidity and postoperative complications.^1^

Estimations on the probability of each adverse event are of paramount importance for both surgeons and patients, as treatment decisions are often based on these estimations.^5^ Several tools have been developed aiming to standardize risk assessment.^6^ Before these risk estimation tools were developed, the decision to treat an aneurysm was solely based on the experience, principles, preferences and views of the treating surgeon. It is unclear how often risk assessment tools are currently used in vascular surgical practice.^6,7^

In 2013 the American College of Surgeons National Surgical Quality Improvement Program (ACS NSQIP) developed the NSQIP risk calculator using data of 780 hospitals. Apart from the planned procedure the NSQIP calculator takes 20 patient variables into consideration in predicting the chance of unfavourable outcomes within the 30-day period after surgery.^5^ Since the development of the NSQIP, no other tool has been developed for the estimation of operative complications which is based on more data.^6^

Only a limited number of studies compared specialists’ probability estimates with the NSQIP risk calculator outcomes. In 2018 Hacohen Solovitz et al.^8^ showed that senior and resident anaesthesiologists unsuccessfully estimated surgical risks based on preoperative data. Sacks et al.^5^ tried to determine if exposure to the outcomes of the NSQIP risk calculator would influence risk assessment of surgeons in acute cases. They reported that exposure to the calculator outcomes based on predefined objective criteria and data would lead to less variance and greater accuracy in postoperative complication estimations.

Following the development of the NSQIP risk calculator, no attempt has been made to compare the NSQIP calculated risks with the risk estimation capacities of different medical specialists involved in complex elective surgeries, more specifically surgical repair of aortic aneurysms.

The aim of this study is to compare the NSQIP risk calculator outcomes with risk assessments by medical specialists, i.e. vascular surgeons, interventional radiologists and anaesthesiologists, involved in treating patients with aortic aneurysms in the Netherlands. Furthermore, we wanted to compare the risk estimations between various specialists.

## Methods

### Study design

An online clinical vignette-based survey study was conducted in which the study population was asked to evaluate five patient vignettes. Patient vignettes are fictitious patient cases which are based on realistic clinical situations and are generally accepted as a tool for measuring the quality of clinical practice. Research has shown that vignette-based survey studies produce better measurement of physician’s decision variation than those based on, often incomplete, medical records. They offer the opportunity for assessments that require patient variables to be modified or for variation to remain constant.^9–11^ For practical purposes the NSQIP risk calculator was perceived as the gold standard of risk stratification tools.

Five clinical vignettes were designed using the methodological recommendations formulated by Evans et al.^11^ The vignettes described patients who were eligible for elective aneurysm repair. Five different procedure types were included in the vignettes; EVAR, fEVAR, bEVAR, tube OSR, bifurcated OSR. The vignettes themselves were designed based on patient factors needed to complete the NSQIP risk calculator; past medical history, medication, smoking, functional status, American Society of Anaesthesiologists physical status classification system (ASA), weight, height, Body Mass Index (BMI) and renal function (Appendix 1). Anatomical and radiological specifications of aortic pathologies (e.g. stenosed access or target vessels, and neck angulation), were left out of the descriptions, as these are not used in the NSQIP risk calculator and could interfere with risk assessments based on systemic and functional impairment.

The participants were asked to give their estimation of the likelihood (on a scale of 0–100%) of postoperative complications and events within thirty days following aortic surgery. Outcome parameters were limited to the most clinically relevant as perceived by the researchers; serious complication, pneumonia, cardiac complication, renal failure, readmission, return to operation room (OR), death and discharge to nursing or rehab facility. Moreover, the study population was asked to give a predicted length of hospital stay in days. The final question asked was whether the specialists ever used a risk stratification tool in clinical situations (never, occasionally or routinely).

‘Serious complication’ was defined according to the NSQIP definition. To be specific: cardiac arrest, myocardial infarction (MI), pneumonia, progressive renal insufficiency, acute renal failure, pulmonary embolism (PE), deep venous thrombosis (DVT), return to the OR, deep incisional surgical site infection (SSI), organ space SSI, systemic sepsis, unplanned intubation, urinary tract infection (UTI), wound disruption. ‘Cardiac complication’ was described in the same way, as cardiac arrest or MI. ‘Renal failure’ was defined as progressive renal insufficiency or acute renal failure. Definitions were available for the participants throughout the survey.

### Participants

The study population consisted of experienced vascular surgeons, interventional radiologists and anaesthesiologists working in assorted hospitals in the Netherlands. In the Netherlands interventional radiologists perform EVAR procedures in conjunction with surgeons in the interventional suite and are involved in preoperative treatment decisions. The participants were recruited via email using snowball and purposive sampling through personal contacts of the research team and by direct solicitation of colleagues from their professional network.^12^

### Data collection

Data was collected through an online survey using a cloud-based data collection service, Castor Electronic Data Capture.^13^ Castor EDC is completely committed to FAIR-data management as well as Good Clinical Practice guidelines. Data will be encrypted, anonymized and, upon request, shared.

### Statistical analyses

The ANOVA test was used to assess significant differences between the risk assessments by the various specialists. The Student t-test was used to analyse significant differences between the NSQIP calculated risks, and the assessments performed by the specialists. Data was processed using SPSS statistics (Version 26, Chicago, IL). *p*-Values of .05 were considered to be significant. Graphs were designed using Graphpad prism (Version 8, San Diego, CA).

## Results

A total of 32 medical specialists agreed to participate in the survey study, i.e. 12 vascular surgeons, 10 interventional radiologists and 10 anaesthesiologists. Table 1 shows a percentage of 68.8% worked in an academic hospital, 21.9% worked in a non-academic hospital and 9.4% chose ‘other’. Half of the participants reported to never have used any risk assessment tool in clinical practice including all of the interventional radiologists, whereas 34.4% occasionally uses a risk assessment tool and 15.6% routinely uses a risk assessment tool.

**Table 1.** Characteristics of study population.

	Vascular surgeons (*n* = 12)	Anesthesiologists (*n* = 10)	Intervention radiologists (*n* = 10)	Total (*n* = 32)
Mean in years (SD)/*n* (%)				
Years of experience	14.33 (6.91)	8.10 (6.71)	13.10 (5.95)	12.00 (6.91)
Practice type				
Academic	6 (50.0)	10 (100.0)	6 (60.0)	22 (68.8)
Private	5 (41.7)	–	2 (20.0)	7 (21.9)
Other	1 (8.3)	–	2 (20.0)	3 (9.4)
Risk assessment tool used				
Never	6 (50.0)	–	10 (100.0)	16 (50.0)
Occasionally	5 (41.7)	6 (60.0)	–	11 (34.4)
Routinely	1 (8.3)	4 (40.0)	–	5 (15.6)

Table 2 illustrates the outcomes of the NSQIP risk calculator per vignette and the mean risk assessments of the participating specialists. Of the total amount of outcome parameters (nine) per vignette multiplied by five vignettes, only five significant differences were found between the risk estimates of different specialist groups. For patient vignette 1, the assessment of risk of death by the anaesthesiologists was significantly lower than those of the interventional radiologists (mean difference (MD) = 2.27, *p* = .048). This was the same for patient vignette 4, where the assessment of risk of death by the anaesthesiologists was significantly lower compared to the vascular surgeons (MD = 13.10, *p* = .019). For patient vignette 3, the interventional radiologist predicted length of hospital stay to be longer compared to the vascular surgeons (MD = 3.65, *p* = .030) and pneumonia more likely to happen compared to the anaesthesiologists (MD = 5.34, *p* = .037). For patient vignette 5, the risk assessment of serious complication by the vascular surgeons was significantly higher than those made by the interventional radiologists (MD = 13.10, *p* = .019). There were no significant differences between groups for patient vignette 2.

**Table 2.** Risk assessment means in % (SD).

Outcomes	Serious complication	Pneumonia	Cardiac complication	Renal failure	Re-admission	Return to OR	Death	Discharge to nursing or rehab facility	Predicted length of hospital stay
Mean in % (SD)/days (SD)									
Patient vignette 1									
NSQIP	9.60	0.60^a^	1.10^a^	0.80^a^	7.00^a^	4.50^a^	0.80^a^	3.30	3.00^a^
Vascular surgeons	10.73 (7.94)	4.71 (2.94)	5.89 (2.82)	5.54 (4.24)	4.56 (4.32)	2.73 (1.43)	3.21 (1.98)	6.11 (8.11)	4.50 (1.51)
Anesthesiologists	6.73 (1.80)	2.61 (2.77)	3.45 (1.68)	3.16 (2.06)	4.35 (2.62)	2.93 (1.66)	1.06 (0.46)	2.80 (1.94)	4.10 (1.37)
Intervention radiologists	10.35 (5.98)	6.47 (4.92)	6.95 (8.53)	6.32 (3.52)	6.78 (5.82)	3.08 (2.02)	3.33 (2.89)	12.43 (27.41)	7.50 (5.46)
Patient vignette 2									
NSQIP	16.70^a^	1.50^a^	2.50^a^	2.00^a^	11.80^a^	5.80^a^	3.30	19.60^a^	4.00^a^
Vascular surgeons	14.35 (5.65)	8.18 (5.65)	8.33 (6.03)	7.97 (7.73)	5.08 (3.69)	3.72 (3.32)	4.29 (3.42)	9.83 (10.26)	5.00 (2.41)
Anesthesiologists	12.99 (4.40)	5.57 (2.83)	4.87 (2.77)	4.03 (2.39)	6.75 (4.19)	3.69 (1.50)	2.7 (1.40)	11.54 (9.04)	6.10 (1.91)
Intervention radiologists	11.47 (9.27)	10.80 (5.63)	7.97 (4.93)	7.84 (4.80)	7.32 (5.85)	5.38 (3.20)	5.27 (3.20)	14.11 (19.17)	9.30 (8.71)
Patient vignette 3									
NSQIP	9.30	1.20^a^	1.10^a^	0.50^a^	8.70^a^	3.90^a^	1.20^a^	5.50	3.50^a^
Vascular surgeons	10.17 (8.44)	4.45 (4.58)	5.21 (3.34)	8.42 (6.10)	3.08 (2.74)	2.19 (1.69)	2.78 (2.90)	4.42 (3.98)	3.25 (1.71)
Anesthesiologists	7.72 (3.99)	2.25 (1.42)	2.37 (0.80)	6.39 (3.07)	4.93 (3.28)	2.88 (1.29)	1.79 (0.69)	6.55 (6.56)	5.10 (1.60)
Intervention radiologists	7.56 (3.86)	7.59 (6.34)	5.72 (4.74)	11.28 (8.09)	4.92 (5.86)	2.96 (3.37)	2.87 (1.90)	8.46 (13.77)	6.90 (5.11)
Patient vignette 4									
NSQIP	21.8^a^	5.10^a^	2.80^a^	3.30^a^	8.00^a^	6.70^a^	2.80^a^	14.20	7.50^a^
Vascular surgeons	18.25 (9.44)	14.04 (9.66)	10.03 (5.88)	5.97 (4.77)	6.62 (4.36)	4.48 (3.66)	5.60 (2.02)	9.55 (9.71)	8.67 (2.06)
Anesthesiologists	15.27 (9.92)	7.03 (3.12)	5.10 (3.67)	4.25 (2.66)	6.69 (5.13)	4.91 (1.89)	3.41 (1.82)	13.93 (10.95)	7.20 (1.03)
Intervention radiologists	12.27 (6.39)	11.39 (6.77)	7.52 (4.59)	6.82 (4.64)	5.03 (4.07)	3.44 (2.21)	3.82 (1.27)	10.43 (14.96)	10.20 (4.49)
Patient vignette 5									
NSQIP	26.4^a^	9.40^a^	4.30^a^	6.20	8.00	11.10^a^	2.50^a^	15.9	7.50^a^
Vascular surgeons	22.99 (14.37)	16.63 (10.15)	10.48 (7.69)	7.28 (4.71)	7.33 (4.57)	4.65 (4.19)	6.25 (2.55)	9.59 (11.66)	8.58 (2.02)
Anesthesiologists	14.57 (9.10)	10.14 (4.50)	4.92 (2.00)	5.75 (3.26)	6.97 (4.88)	5.22 (3.24)	4.15 (2.86)	15.87 (11.50)	8.60 (2.68)
Intervention radiologists	9.89 (4.90)	13.25 (13.61)	6.40 (5.44)	6.39 (6.66)	7.68 (9.73)	4.05 (2.67)	4.38 (1.68)	10.06 (16.55)	10.80 (5.43)

^a^Significantly different from the combined risk assessments of the specialists.

**Figure 1.**
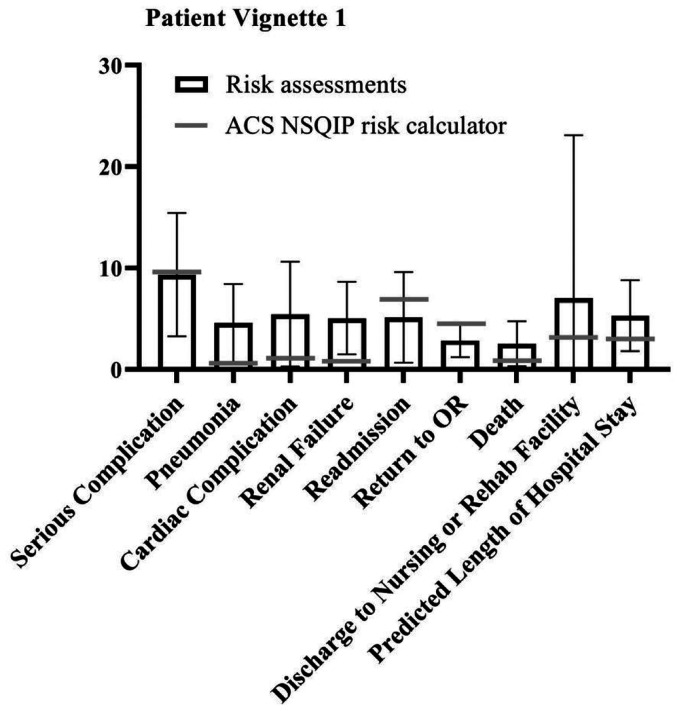
Overview of risk assessments patient vignette 1.

**Figure 2. fig2-20480040211006582:**
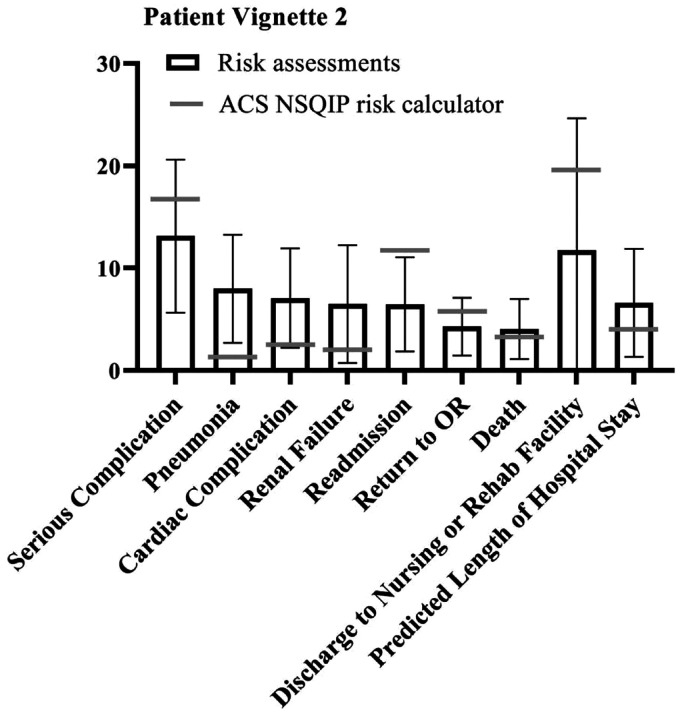
Overview of risk assessments patient vignette 2.

**Figure 3. fig3-20480040211006582:**
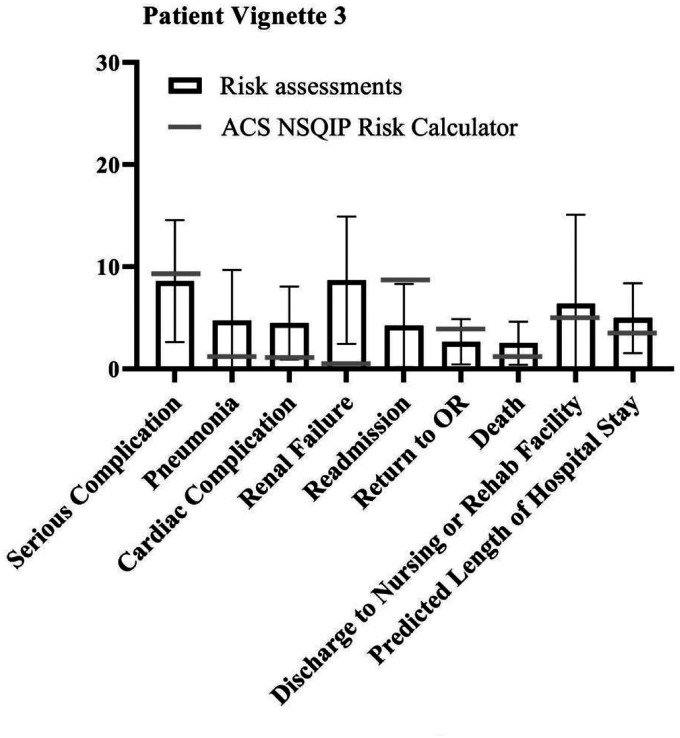
Overview of risk assessments patient vignette 3.

**Figure 4. fig4-20480040211006582:**
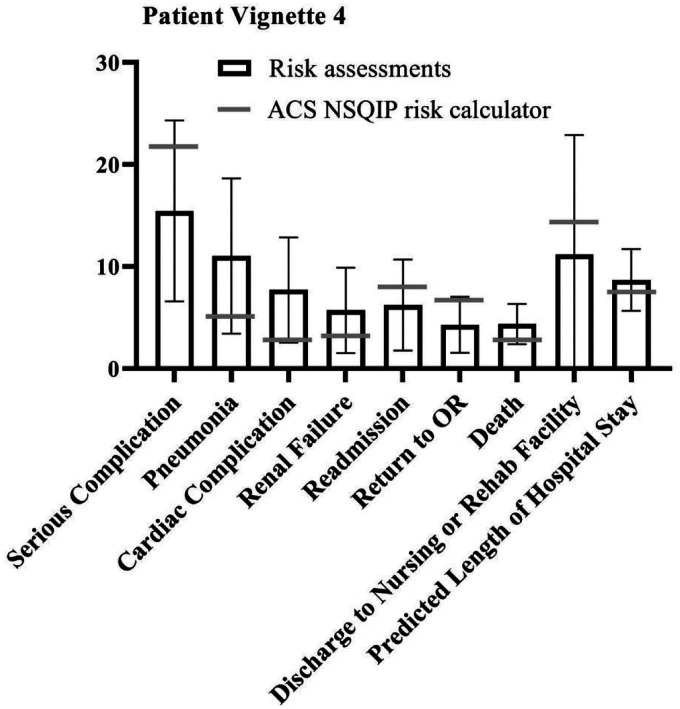
Overview of risk assessments patient vignette 4.

Since few outcome parameters showed significant differences between different specialties, further statistical tests were performed on the study population as one group (vascular surgeons, anaesthesiologists and interventional radiologists combined). The student t-test showed a significant difference between the NSQIP calculator outcomes and the combined risk assessments of the specialists in most outcome parameters in all patient vignettes. However, in four of the five vignettes, excluding patient vignette 2, the specialists accurately predicted the risk of discharge to rehab of nursing facility compared to the risk calculator (*p* = .196, *p* = .587, *p* = .156 and *p* = .082 for patient vignette 1, 3 through 5 respectively). ‘Serious complication’ was accurately predicted by the specialists for patient vignettes 1 (*p* = .827) and 3 (*p* = .505). For patient vignette 2, specialists were closest to the risk calculator outcome regarding death (*p* = .137). When assessing risks for patient vignette 5, the specialists were most accurate for renal failure (*p* = .714) and readmission (*p* = .560). Overall, specialists assessed the risks significantly different compared to the NSQIP risk calculator, but neither particularly higher nor lower. Figures 1 [Fig fig2-20480040211006582][Fig fig3-20480040211006582][Fig fig4-20480040211006582]to 5 show per vignette an overview of the risk assessments of the specialists and the calculated risks.

**Figure 5.**
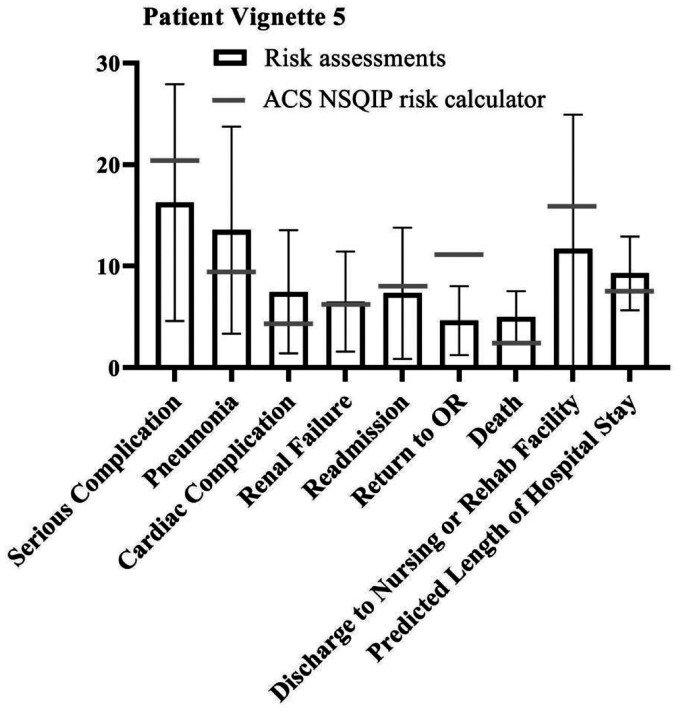
Overview of risk assessments patient vignette 5.

When comparing the risk assessments based on ‘risk calculator use’, no significant differences were found for patient vignette 1, 4 and 5. For patient vignette 2, the risk assessment of pneumonia by the specialists who never used the risk calculator was higher than those made by the specialists who used the tool routinely (MD = 6.04, *p* = .046). Risk of death for patient vignette 2 was also assessed to be higher by the group who never used the calculator compared to the group who used the tool occasionally (MD = 2.92, *p* = .026). This was the same for the risk assessment of pneumonia for patient vignette 3 relative to occasional use (MD = 4.48, *p* = .047). Comparing these risk assessments to the NSQIP risk calculator outcomes using the student t-test showed that the specialists who used the calculator routinely were the most accurate. That is, for patient vignette 2 for risk of pneumonia (p = .105), risk of death (*p* = .505) and for risk of pneumonia for patient vignette 3 (*p* = .277).

## Discussion

This survey study compares the risk assessments between vascular surgeons, interventional radiologists and anaesthesiologists involved in aortic aneurysm repair. Subsequently, the risk assessments of the aforementioned specialists were compared with the NSQIP risk calculator outcomes. We found that the specialists differed significantly in almost every case from the risks calculated with the NSQIP risk calculator.

There are various reasons to assess risk prior to a surgical procedure. Firstly, it can be used to better inform the individual patient and to permit solid informed consent. Secondly, risk assessment can potentially help reduce risk through preoperative counselling and work-up. Finally, standardized risk estimates can improve postoperative management in offering a stronger basis for preoperative discussion and planning among treatment teams. Therefore, standardized risk stratification can potentially reduce costs, staff and facility usage such as admission to the intensive care unit and total length of hospital stay. It can, additionally, enable comparison of outcomes of identical procedures between different surgeons or hospitals.^6,8,14^

Two studies address the role of the NSQIP calculator in clinical preoperative decision-making. Sacks et al.^5^ reported that surgeons would not change their decision to operate after exposure to the NSQIP risk calculator outcomes. No significant difference in likelihood of recommending an operation between surgeons in the control group and the group exposed to the calculated risks were reported. However, surgeons exposed to the risk calculator outcomes made estimates closer to these outcomes and varied less compared to the control group. In contrast, our study reports no difference in estimation between specialists who used the risk calculator routinely in their clinical practice compared to specialists who used the tool occasionally or never. This could be explained by the fact that our study population did not have the calculator at their disposal during the survey and the relatively low number of participants.

Hacohen Solovitz et al.^8^ compared NSQIP risk calculator outcomes to estimates made by resident and senior anaesthesiologists. They showed that both groups did not predict the various complications with much accuracy compared to the NSQIP calculator, which is similar to the outcomes of our study. It should be emphasized that the data were not compared to actual clinical outcomes, therefore the present study does not consider the risk assessments of the medical specialists to be true or false.

Numerous studies compare the NSQIP risk calculator values to clinical outcomes.^15–21^ These studies show that the risk calculator is an adequate method to predict risks of postoperative complications, however it comes with some disadvantages. Notably, it is currently not possible to incorporate specific risk factors for individual procedures. For example, in a case of aortic aneurysm surgery, the number of fenestrations, aortic angles and thrombus or stenosis of the target vessels cannot be specifically taken into account. Consequently, the risks of ‘procedure specific complications’ are not provided by the risk calculator. One might say that the complexity and the likelihood of success of the procedure are for a large proportion determined by anatomical features, undermining the relevance of this risk calculator.^22^ Maintaining a distinction between procedure specific and other risks is, however, well established in literature. Systemic complications in relation with patient specific factors without looking at anatomical features other than aneurysmal diameter is well described.^23–26^ Nejim et al.^23^ reported predictive factors besides aneurysmal diameter, for in-hospital adverse events after elective EVAR. These included advanced age, female sex, hypertension, chronic obstructive pulmonary disease, congestive heart failure, coronary artery disease, chronic kidney disease, diabetes mellitus, obesity and smoking.

The studies that examine the effect of anatomical factors often only explore a correlation between procedure specific adverse events or survival, frequently after the 30-day postoperative period.^27–33^ For instance, Oliveira et al.^33^ showed that an aortic diameter >70 mm is an anatomical independent risk factor for an increase in late all-cause mortality after EVAR, whereas reverse-tapered neck configuration, angle, neck thrombus, neck calcification, infrarenal neck diameter and length are not. Moreover, they found that age, ASA ≥ 3 and renal insufficiency are correlated to decreased survival. All this might imply that morphological features, except aneurysm diameter, do not have a large impact on complications in general, especially within thirty days postoperatively.

In the current study morphology was deliberately left out of consideration because of the high impact of systemic patient factors on non-procedure related adverse events and the relatively low evidence of the correlation between anatomical features and short-term systemic outcomes. Moreover, specific anatomical information might also distort the judgement of the specialists regarding general adverse events and render the results not comparable with the NSQIP results, which was the main purpose of this study. In addition, anaesthesiologists often look exclusively at patient systemic data, using the ASA classification system and leave the anatomical factors out of their risk estimations altogether.^34^ The underlying intention of this study was to determine whether the NSQIP risk calculator ought to be incorporated in a preoperative multidisciplinary approach of assessing a patient for aneurysm repair. In this manner, a combined risk assessment (both procedure and non-procedure related) can be made more accurately and can be discussed with a patient.

Currently, the Society for Vascular Surgery recommends the use of the vascular quality initiative (VQI) algorithm. This algorithm, developed in a cohort of 8000 patients, predicts the likelihood of postoperative myocardial infarction, congestive heart failure, or arrhythmia requiring treatment.^35^ Compared to NSQIP risk calculator the VQI algorithm is slightly more procedure specific, taking aneurysmal diameter into consideration for elective EVAR, clamp positioning and level of distal anastomosis for OSR. However, the number of outcomes calculated are limited to a cardiac risk index, whereas the NSQIP risk calculator delivers a much broader assessment of possible complications relevant for multidisciplinary discussion and planning.

Another concern regarding the NSQIP calculator is that it was developed with American patient data and has not yet been validated outside of the US, which implies the necessity of a validation study. Furthermore, the ASA classification system is incorporated in the NSQIP calculator, while studies show that there may be significant inter-rater variability in the ASA classification system.^14^ Nevertheless, the NSQIP risk calculator clearly shows potential. In a recent review of available risk stratification tools, it is currently recognized as being the best in the field and further development of the calculator is continuous.^7^

This study has some limitations. Firstly, the size of our study population is limited (n=32). This could be an explanation for the fact that we found little significant differences between specialists? risk assessments. Secondly, the unequal distribution of the study population between the different types of clinical practice (all anaesthesiologists worked in an academic setting) together with the differences in experience with a risk assessment tool (interventional radiologists had no experience using the NSQIP risk calculator) could lead to bias. However, all included medical specialists have experience treating complex aortic pathology (both academic and non-academic) and are familiar with risk assessment (with or without the use of risk calculating tools). Thirdly, snowball and purposive sampling was used, which, although a recognized and accepted selection technique, could be considered as a risk of bias. Fourthly, due to the use of fictional patient vignettes it is not clear whether the results of the current study would extend to actual clinical practice. Lastly, the assessments made by the specialists and those generated by the NSQIP risk calculator were not compared with actual outcomes also affecting generalization of results.

Our findings however show the lack of consensus and a need for the implementation of an objective risk stratification tool, such as the NSQIP risk calculator, for multidisciplinary preoperative risk assessment. Phrasing a definite statement regarding the need for the implementation of the NSQIP risk calculator remains an ongoing process.

## Conclusions

This study found that various specialists estimate perioperative risks within the framework of both endovascular and open aortic aneurysm repair significantly different when compared to the NSQIP risk calculator. Based on these results, the NSQIP risk calculator should be implemented in preoperative multidisciplinary team meetings, patient information and patient planning.

## Appendix 1. Patient vignettes.

### Vignette 1

An elective **bEVAR** will be performed on a 66-year-old-male who lives with his wife and has two daughters. He works as a history teacher and loves to ski.

**Past medical history:** Spiral tibial shaft fracture, PCI with 4 Drug-eluting stents RCA

**Medication:** carbasalate calcium, statin, metoprolol

**Intoxications:** none

**Functional Status:** independent

**ASA:** 3

**Weight:** 85kg; **Height:** 170cm; **BMI:** 29.5kg/m^2^

**Renal function:** creatinine 87µmol/L, eGFR 79

### Vignette 2

An elective **fEVAR** will be performed on a 78-year-old male. He formerly worked as a truckdriver.

**Past medical history:** CVA with residual deficit, hypercholesterolemia, hypertension, LUTS

**Medication:** atorvastatin, clopidogrel, perindopril, tamsulosin

**Intoxications:** stopped smoking 4 years ago (smoked 10 cigarettes a day for 58 years), drinks 3 to 5IE alcohol a day

**Functional status:** partially dependent

**ASA:** 3

**Weight:** 127kg; **Height:** 192cm; **BMI:** 34.45kg/m2

**Renal function:** creatinine 67µmol/L, eGFR 88

### Vignette 3

An elective **EVAR** will be performed on a Spanish 75-year-old widow who has 3 sons and previously worked as a housekeeper.

**Past medical history:** appendectomy, kidney transplant recipient

**Medication:** cyclosporine, azathioprine, prednisone, carbasalate calcium, statin

**Intoxications:** stopped smoking 10 years ago (smoked 10 cigarettes a day for 20 years)

**Functional status:** independent

**ASA:** 3

**Weight:** 73kg; **Height:** 173cm; **BMI:** 24.4 kg/m2

**Renal function:** creatinine 123µmol/L, eGFR 42

### Vignette 4

An elective **open tube graft insertion** will be performed on a 75-year-old male who was successfully treated for metastasized (liver) colon cancer 9 months ago.

**Past medical history:** hypertension, diabetes mellitus type 2, metastasized (liver) colon cancer, atrial flutter, TURP regarding prostatitis.

**Medication:** enalapril, metformin, carbasalate calcium, statin, omeprazole, paracetamol, tadalafil, zolpidem

**Intoxications:** smokes 19 cigarettes a day, social drinker

**Functional status:** independent

**ASA:** 2

**Weight:** 81kg; **Height:** 180cm; **BMI:** 25.0kg/m2

**Renal function:** creatinine 86µmol/L, eGFR 85

### Vignette 5

An elective **open bifurcation graft insertion** will be performed on a 69-year-old male who previously worked as chef. There is a positive family history of mental illness and several types of cancer.

**Past medical history:** alcohol abuse, hypercholesterolemia, hypertension, COPD gold 3, basal cell carcinoma

**Medication:** valsartan, hydrochlorothiazide, metoprolol, pantoprazole, simvastatin, formoterol/beclometason, tiotropium, carbasalate calcium

**Intoxications:** stopped smoking 6 months ago (smoked 19 cigarettes a day for 50 years), drinks 4-8IE a day

**Functional status:** independent

**ASA:** 3

**Weight:** 104kg; **Height:** 181cm; **BMI:** 31.7kg/m2

**Renal function:** creatinine 69µmol/L, eGFR 88
